# Alterations of medial prefrontal cortex bioelectrical activity in experimental model of isoprenaline-induced myocardial infarction

**DOI:** 10.1371/journal.pone.0232530

**Published:** 2020-05-08

**Authors:** Marko Vorkapić, Andrej Savić, Milica Janković, Nemanja Useinović, Milica Isaković, Nela Puškaš, Olivera Stanojlović, Dragan Hrnčić

**Affiliations:** 1 Institute of Medical Physiology “Richard Burian”, Faculty of Medicine, University of Belgrade, Belgrade, Serbia; 2 University of Belgrade–School of Electrical Engineering, Belgrade, Serbia; 3 TECNALIA, Health Division, Donostia-San, Sebastian, Spain; 4 Institute of Histology and Embryology “Aleksandar Đ Kostić” Faculty of Medicine, University of Belgrade, Belgrade, Serbia; Technion Israel Institute of Technology, ISRAEL

## Abstract

**Background:**

Clinical and animal studies have found that anxiety and depression are significantly more common after acute myocardial infarction (AMI). The medial prefrontal cortex (PFC) has a dual role: in higher brain functions and in cardiovascular control, making it a logical candidate for explaining the perceived bidirectional heart-brain connection. We used parallel Electrocardiography (ECG) and Electrocorticography (ECoG) registration to investigate AMI-induced changes in medial PFC bioelectrical activity in a rat model of AMI.

**Materials and methods:**

Adult male Wistar albino rats were used in the study. Gold-plated recording electrodes were implanted over the frontal cortex for ECoG recording. ECG was recorded via two holter electrodes attached on the skin of the back fixed in place by a jacket. Induction of AMI was performed by isoprenaline (150 mg/kg, i.p.). ECoG and ECG signals were registered at baseline, during 3 hours after isoprenaline administration and at 24 hours after isoprenaline administration.

**Results:**

Significant increases of theta, alpha, and beta electroencephalographic (EEG) band power were observed in different time intervals after isoprenaline administration. Significant increase of theta band peak frequency was also observed during the first hour after isoprenaline administration. No statistically significant differences in band-power activity were found between the pre-isoprenaline measurements and 24 hours after administration.

**Conclusion:**

Our results demonstrate significant increases in EEG band power of alpha beta and theta bands during isoprenaline-induced AMI model. These are the first findings to connect heart damage during isoprenaline- induced AMI to disturbances in the cortical bioelectrical activity.

## Introduction

Although there are well-defined risk factors for cardiovascular diseases (CVD) (elevated blood levels of cholesterol, hypertension, diabetes mellitus, smoking etc.) [1], they cannot explain the full scope of variability in presentation and prognosis. Therefore, significance of bidirectional relationship between psychological, neurological and autonomic nervous factors and heart diseases is becoming more recognized [2].

Large case control studies, such as the INTERHEART study, have shown psychosocial stressors are related to higher risk of heart diseases such as acute myocardial infarction (AMI) [3]. Also, connection of CVD, especially AMI, and mood disorders, primarily depression, has been well documented [4–6]. Even cognitive decline can be attributed to CVD since it is significantly faster in elderly patients with heart failure and EEG studies have shown changes similar to those in Alzheimer’s disease [7]. While these and many other studies show the epidemiological link of CVD and brain-related disorders in clinical settings, there are only a limited number of experimental studies in animal models exploring the influence of CVD and AMI on brain function.

So far, experimental studies in rodents have shown that acute damage to the myocardium, causes neuronal death, affective disorders and disruption of the circadian rhythm. Namely, within 72 hours of AMI onset there is neuronal loss in the central nervous system (CNS), most importantly in the lymbic system, hypothalamus and brainstem [8,9]. Further, depression onset after AMI was corroborated by studies in rats looking at behavioral signs of anhedonia and despair 2 weeks post AMI that can be reversed by antidepressants [10–12]. Exploring sleep-related disturbances in rats two weeks post AMI revealed decreased slow wave sleep and decreased latency to rapid eye movement (REM) sleep onset previously recognized in patients with depression [12]. Finally, autonomic disturbance with sympathetic overdrive was detected by heart rate variability (HRV) analysis in the quiet sleep (QS) stages and can account for some of the sudden cardiac deaths occurring due to arrhythmias in the late faze post AMI [13]. All of the above-mentioned studies focus on changes during the chronic faze post AMI, and we are currently lacking in literature exploring the imminent faze of ischemia onset.

Also, there have been no studies examining the effects of AMI on cortical areas known to be involved in cardiovascular regulation: the insular cortex (IC) [14–16] and the medial PFC [17–19]. The medial PFC is considered as more involved in higher brain functions: executive control, memory, decision making, social interactions etc. However, its effects on heart regulation have recently become more recognized [20]. If found, perturbations in function of the medial PFC caused by AMI could provide the next step in understanding the effect of heart disease on higher brain functions. A possible way to assess the activity of medial PFC neurons is electrocorticograhy (ECoG): a method of signal acquisition using electrodes implanted directly on the brain surface [21]. This method enables us to follow the changes in neuronal activity in real-time during the acute faze of AMI in parallel with ECG monitoring. In exploring the connection of medial PFC function and heart disease we used the experimental model of isoprenaline induced AMI. Isoprenaline is a peripherally acting beta-adrenergic agonist with short plasma half-life known for its cardiostimulatory and bronchodilatatory effects and the isoprenaline model is a well-established model of AMI [22].

Our hypothesis is that ECoG recordings over the medial PFC can reveal changes in bioelectrical brain activity furthering understanding the influence of AMI on cortical function. Therefore, the aim of this study was to assess spectral characteristics of rat ECoG signal registered over medial PFC during isoprenaline-induced AMI in rats.

## Materials and methods

### Animals

All experimental procedures were in full compliance with the European Council Directive (2010/63/EU) and approved by the Ethical Committee of Belgrade University Faculty of Medicine and national ethical body for animal welfare (Permissions No 4455/2 and No 323-07-08097/1/2018-05). All surgery was performed under sodium pentobarbital anesthesia, and all efforts were made to minimize suffering. Adult male Wistar albino rats (2 months old, 200–230 g body weight (b. w.)) were used in the study (obtained from the Military Medical Academy Breeding Laboratory, Belgrade, Serbia). The animals were housed in transparent plastic cages with *ad libitum* access to food and water. They were kept in a sound-attenuated chamber under controlled ambient conditions (22–23°C, 50–60% relative humidity, 12/12 h light/dark cycle with light switched on at 8 a.m.) and habituated to handling. The acclimatization period lasted for 7 days.

### Electrodes placement for EEG and ECG registration

The rats were anesthetized with pentobarbital sodium (50 mg/kg, i.p.). One gold-plated recording electrode was implanted over the frontal cortex in a stereotaxic apparatus. Dental acrylic cement was used to fix this system to the skull. Animals had a recovery period of one week prior to further experiments. A 24-h-long habituation to the recording situation was also applied.

One day before ECG recording, the dorsal thoracic region of each animal was carefully shaved. A custom-made elastic cotton jacket was developed to fit the rat’s mean thoracic circumference. Two holter electrodes with adhesive gel were attached on the skin of the rat for ECG recording, with each electrode being connected to a cable, long enough to reach the acquisition system. The rat was then placed in the jacket fixing the electrodes in place. A 24-h-long habituation to the recording situation was applied.

### AMI induction

In order to secure scientific and statistical validity of this exploratory study, present sample size (n = 12), i.e. number of animals in the groups (n = 6), has been determined according to sample size method of “rescue equitation” analysis [23, 24]. A power analysis shows that the current sample size has an 80% power to detect an effect size of 20% assuming a 5% significance level and a two-sided test. Animals were randomly divided into two equal groups: experimental (n = 6) and control group (n = 6). In the experimental group induction of AMI was performed through intraperitoneal administration of isoprenaline (isoproterenol hydrochloride, Sigma Aldrich, USA) at a dose of 150 mg/kg diluted in 2 ml of saline. The control group was injected with 2ml of isotonic saline.

### Data acquisition

**Fig 1:**
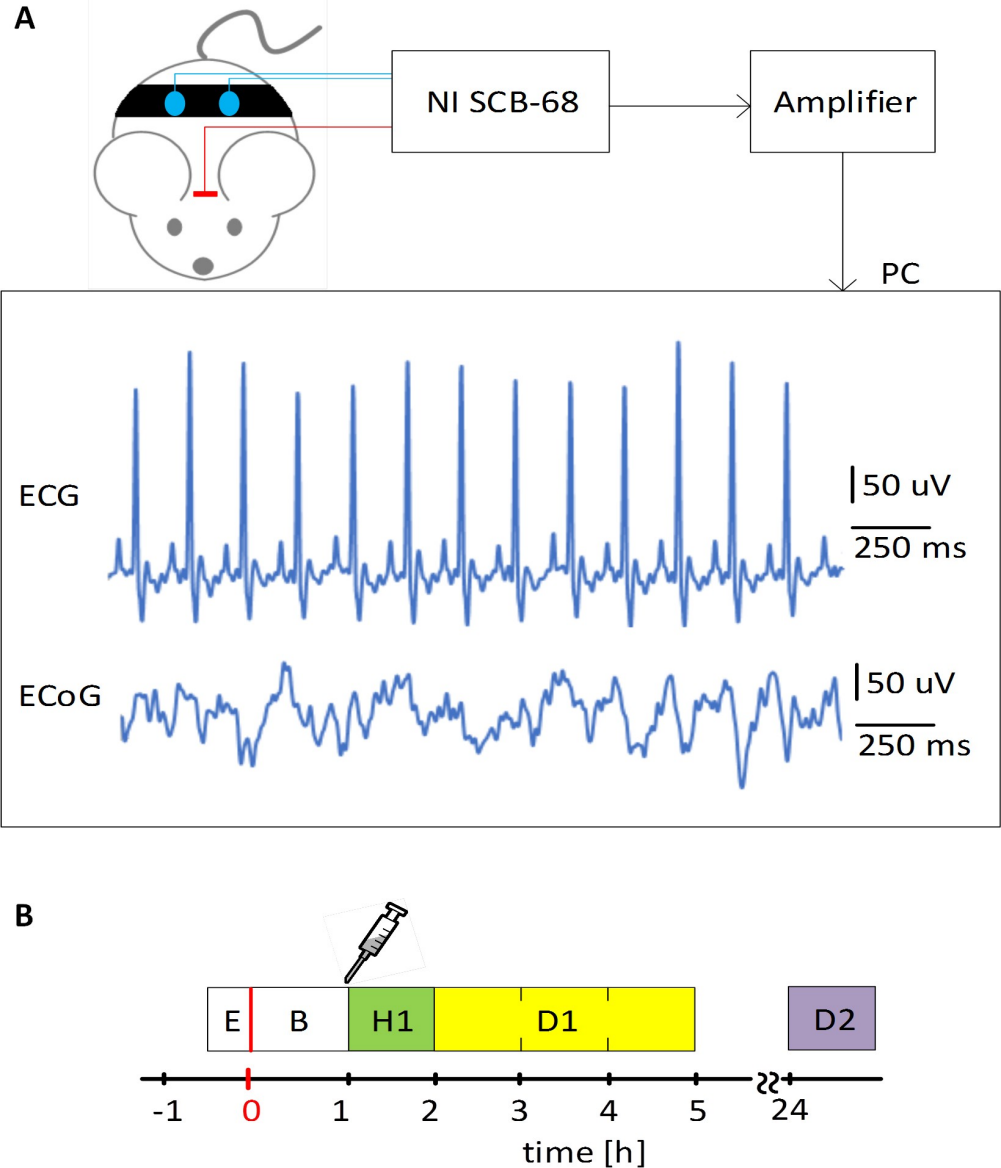
Graphical representation of acquisition setup and timeline. A) Data acquisition setup B) Data acquisition timeline: E–montage of ECG electrodes, B–baseline, H1 –first hour of data acquisition after infarction induction, D1 –second to fourth hours of data acquisition after infarction induction, D2 –one hour of data acquisition in the second day (24h after isoprenaline administration).

Simultaneous data acquisition of ECoG and ECG signals was performed by an EEG amplifier (RIZ, Zagreb, Croatia). The cutoff frequencies of analog filters were set to 0.3 Hz and 100 Hz for the high-pass and low-pass filters, respectively. Notch filter at 50-Hz, for removing the ambient noise, was also applied. Signals were digitized using a 16-bit NI-SCB-68 data acquisition card (National Instruments, Austin, Texas, USA). A sampling frequency was set to 512 Hz. Data acquisition and signal processing were performed using a custom made application NeuroSciLaBG developed in NI LabVIEW software package (National Instruments, Austin, Texas, USA). ECoG and ECG signals were recorded in freely moving rats during a five-hour recording session in the first day (one hour before and four hours after infarction induction) and one hour in the second day (24h after isoprenaline administration). This is illustrated in Fig 1 with denominated periods referred to in the rest of the manuscript. Signal traces were visually inspected and analyzed during subsequent offline analysis.

### Heart tissue sampling and histopathological evaluation

Twenty-four hours after the intraperitoneal injection, the rats were anesthetized with pentobarbital sodium (50 mg/kg, i.p.). The thorax was accessed by an inverted T incision and the heart released by cutting the great vessels of the corona cordis. Hearts were removed from the rat body, fixed in 10% buffered formalin, dehydrated in ethanol, cleared in xylene, and embedded in paraffin. Five μm thick sections were subjected to routine staining with hematoxylin and eosin (HE) and examined under the OlympusBX41 light microscope with OlympusC5060A-ADU digital camera.

### Data processing

**Fig 2:**
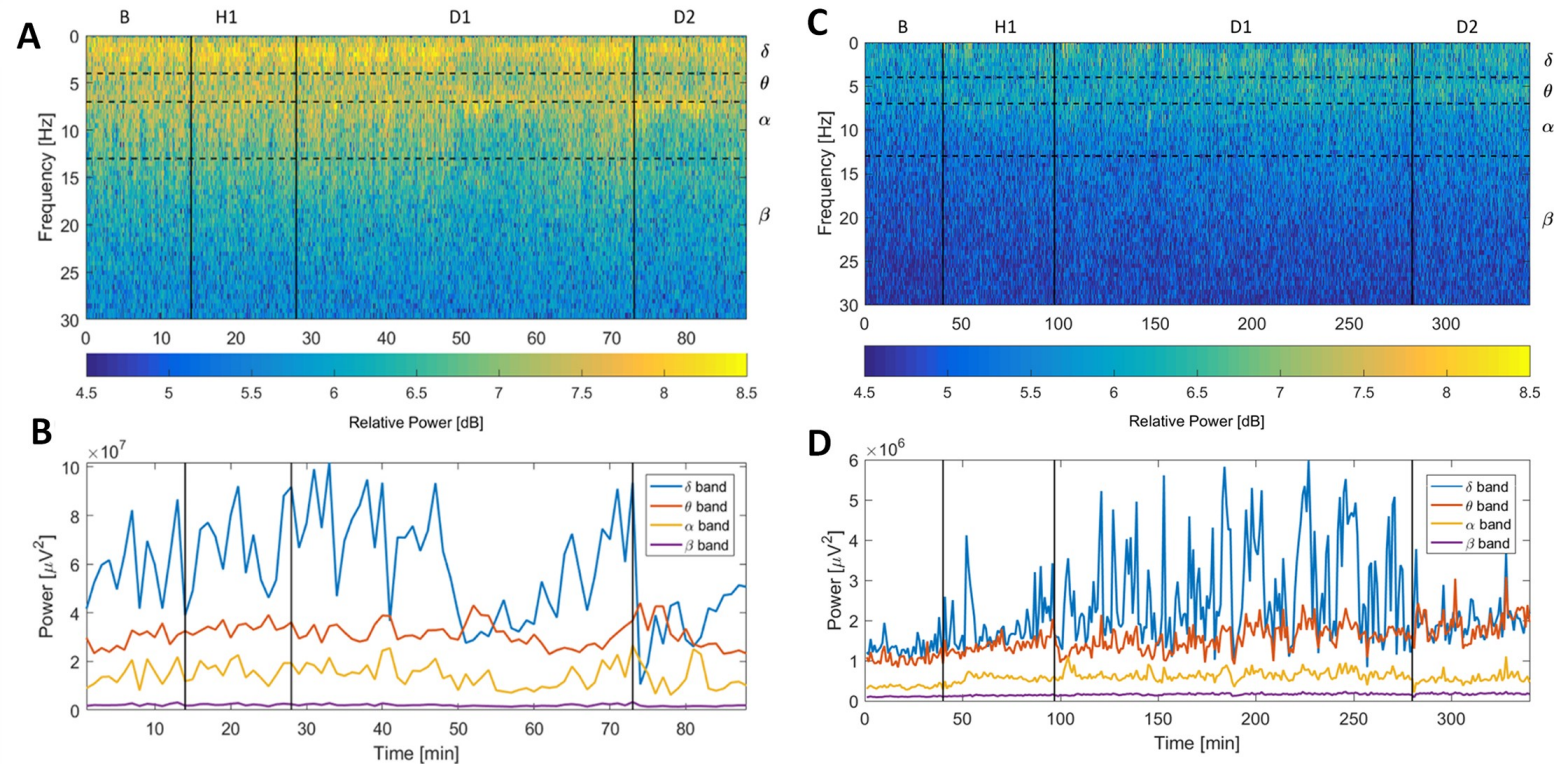
Representative spectrogram and band power graphs. Spectrogram (A, B) and corresponding power changes in delta, theta, alpha and beta frequency bands (C, D) during 4 time-windows for one animal in the control group (left) and experimental group (right).

Continuous EEG data was segmented into non-overlapping 60 second epochs. Segmented data was automatically scanned for determining epochs contaminated with noise. All epochs exceeding 500 μV thresholds were marked as noisy and rejected from further analysis. The rest of the data was visually inspected for artifacts and further epoch-exclusions were applied when necessary. For visual inspection EEG signals' Power Spectrograms were calculated using Short time Fourier Transform. Parameters for Spectrogram calculation were: window width of 2 seconds, window overlap of 0.5 seconds and Hann window function. Representative Power Spectrogram of the rat XX is presented in Fig 2, panel (A).

Band-power time curves for each rat were calculated as the mean power value of the Spectrogram data in the 4 predefined frequency bands: delta (0.5–4 Hz), theta (4–7 Hz), alpha (7–13 Hz) and beta (13–35 Hz). Representative band-power time curves calculated for the spectrogram data of rat xx are presented in Fig 2, panel (B).

For further analysis, The EEG data was segmented into four time windows (as shown in Fig 1): 1) baseline (B), 2) first hour of data acquisition after AMI induction (H1), 3) second to fourth hours after AMI induction (D1), 4) 24h after isoprenaline administration (D2).

Median value of band power for each animal, time window and frequency band were calculated.

Additionally, the EEG epoch peak frequency for each frequency band of interest was defined as a frequency value (Hz) for which the maximum value of the Power spectral density estimate appears within the predefined frequency band limits. The Power spectral density estimate for each epoch was obtained by using Welch algorithm on raw EEG data epochs of 1 second duration. For each animal and time window (B, H1, D1 and D2) we evaluated the median peak frequency, by calculating the median over peak-frequency values of all non-overlapping 1 second epochs within each time window (B, H1, D1 and D2).

Continuous ECG recordings were inspected offline for determination of ST elevation over the course of experiments (time windows B, H1, D1 and D2). Peak ST segment elevation amplitude was determined for each rat in the group according to previously reported study of isoprenaline-induced AMI [25, 26].

Relationship between relative change of median power for total and each EEG frequency band in time windows H1, D1 and D2 versus B (determined as (X-B)/B, where X = H1, D1 or D2) to ST elevation has been investigated by correlation statistics.

### Statistical analysis

Statistical analysis was conducted to compare median band-power values and median peak-frequency values of 4 time-windows of interest.

Wilcoxon’s signed rank test was performed for pairwise comparison between time-windows of interest for each power band. Pearson correlation coefficient was computed to assess the relationship between ST elevation and EEG-derived variables. The significance threshold was set to 0.05.

## Results

### Spectral power

**Fig 3:**
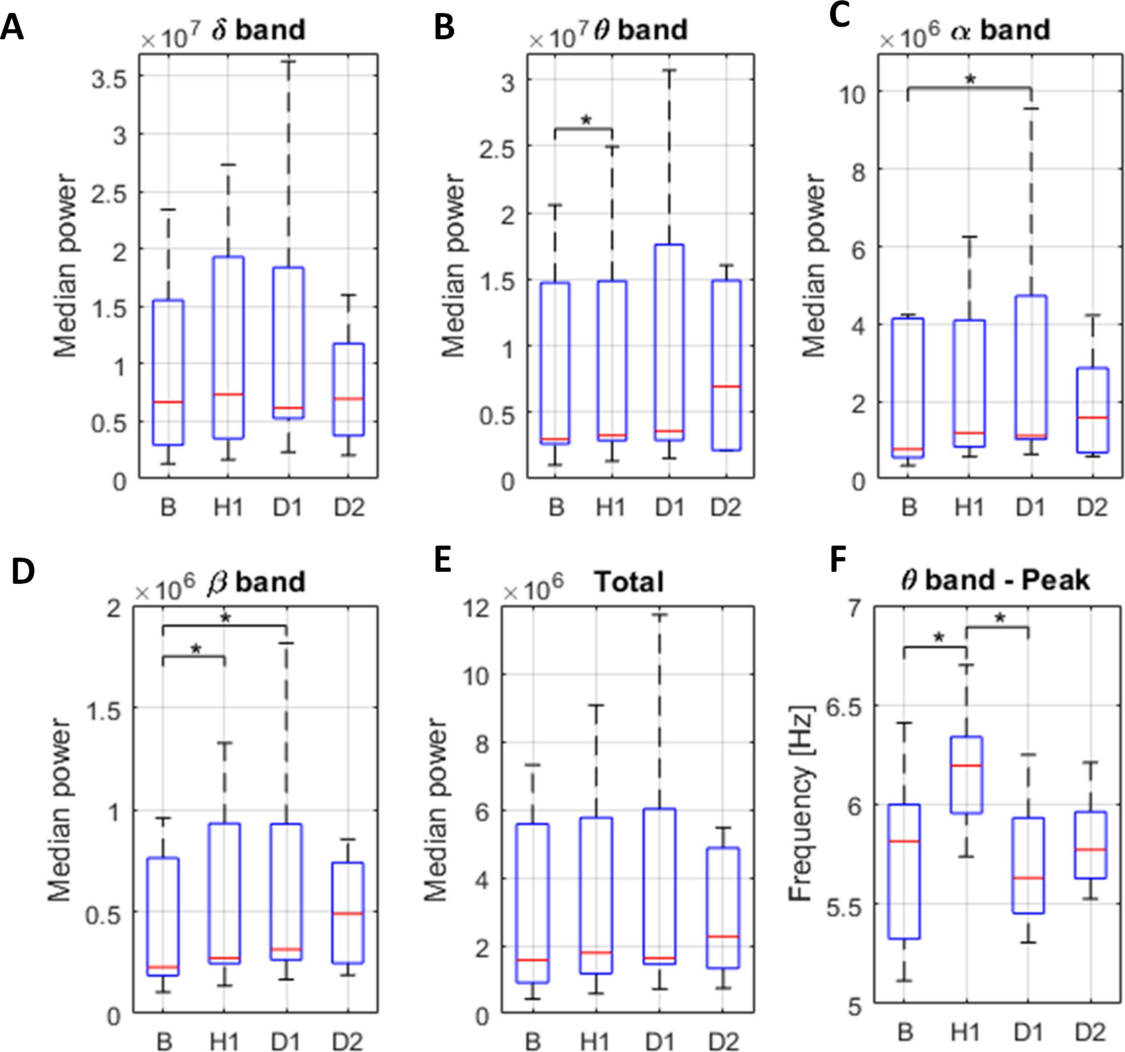
Median power changes per frequency band for the experimental group. Median power of delta (A), theta (B), alpha (C) and beta band (D) and total median power (E). Peak frequency for theta band (F). *p<0.05.

Results of the median power analysis in 4 time-windows for the experimental group is presented in Fig 3.

Statistically significant differences (p<0.05) in median band-power (over all animals) were found in theta, alpha and beta frequency bands. For theta band a statistically significant power difference was found between B and H (p<0.05, Fig 3B); for alpha band between B and D1(p<0.05, Fig 3C); and for beta band between B and H1, and B and D1 (p<0.05, Fig 3D). The observed differences imply significant increase of median band power compared to the baseline, over all animals, in the first hour after isoprenaline administration for theta band, rest of the day 1 for alpha band and both time intervals of day 1 for beta band. No statistically significant differences were found between baseline activity and activity in day 2 for any of the frequency bands, indicating that the obtained differences in time-windows of day 1 were short term effects associated with isoprenaline administration.

Results of the band peak-frequency analysis of the 4 time-windows revealed statistically significant differences of theta band peak frequency. Results of band peak-frequency analysis for theta band are presented in Fig 3F. The results showed significant increase of median theta peak frequency in the first hour after isoprenaline administration compared to the baseline. This effect is not present during the rest of the day1 and day2 windows in which the theta peak frequency did not differ significantly from the baseline. No differences were found in peak frequency analysis in the remaining frequency bands.

**Fig 4:**
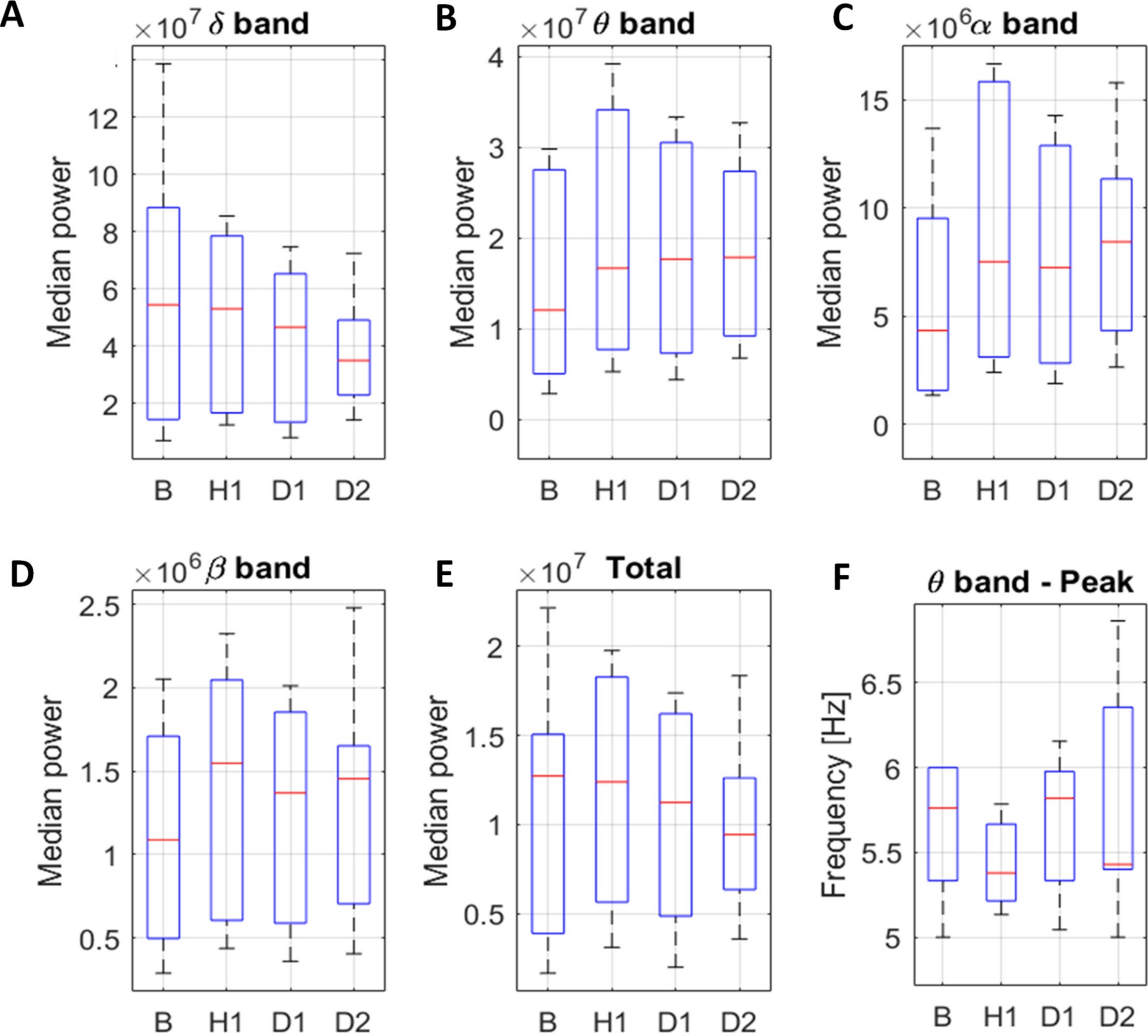
Median power changes per frequency band for the control group. Median power of delta (A), theta (B), alpha (C) and beta band (D) and total median power (E). Peak frequency for theta band (F). *p<0.05.

No differences in median band power were found in any of the observed time-windows in the control group (Fig 4).

### ECG registration

**Fig 5:**
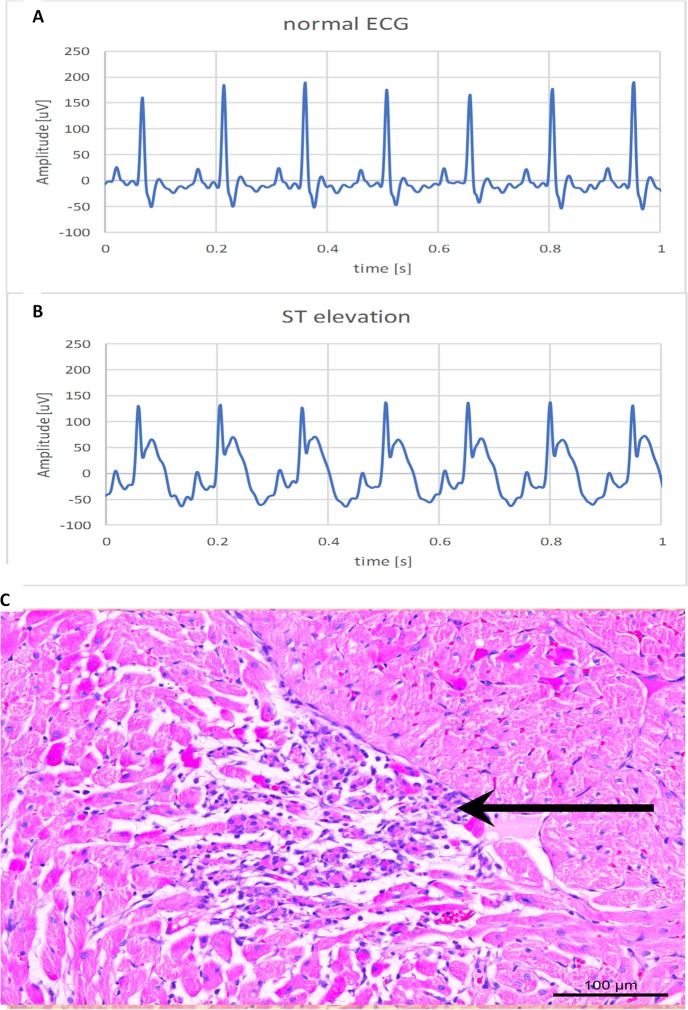
ECG and histological changes after isoprenaline administration. (**A**) Normal ECG trace, **(B**) ECG containing ST elevation, (**C**) Histological changes in the myocardial wall 24 hours after i.p. application of isoprenaline. In the central part of the picture we can notice necrotic cells with hyper-eosinophilia, pyknotic nuclei, and inflammatory cell infiltration (arrow). The ischemic area is surrounded by viable myocardial cells.

ECG recordings in baseline conditions in both experimental and control group revealed no abnormalities in ECG wave morphology and dynamics. However, ST elevation was noted in the experimental and not the control group after 14 minutes and lasted until the end of the first day (representative ECG trace containing ST elevation in Fig 5).

### AMI verification

ST elevation was observed in the experimental group approximately 14 minutes after isoprenaline administration. This was the early marker used to confirm successful AMI induction.

Histological analysis was used as the definitive method in establishing the successfulness of AMI induction. It was performed 24 hours after isoprenaline administration. The slides revealed ischemic injury in the myocardial wall 24 hours after application of isoprenaline in all the rat hearts, while there were no changes in the myocardium of the control (saline treated) group. Ischemic injury is characterized by presence of necrotic myocardium with an interface of inflammation. In Fig 5 we can see a necrotic area with hyper-eosinophilia, pyknotic nuclei, and inflammatory cells infiltration.

### Correlation analysis

**Fig 6:**
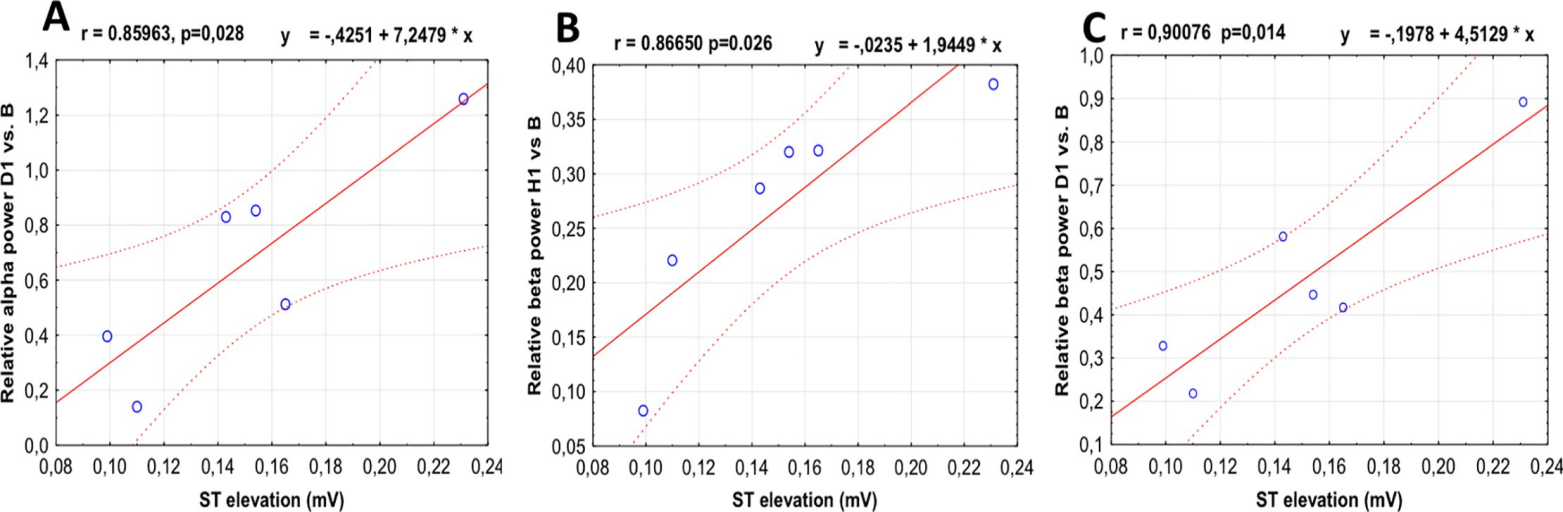
Correlation between ST elevation and EEG-derived variables. According to Pearson correlation analysis, there were a strong positive correlation between the ST elevation amplitude and: relative change in alpha power in D1 (A); beta power in H1 (B) and D1 (C) versus B time window. For details see caption to Figs 1 and 2.

A Pearson correlation coefficient was computed to assess the relationship between the ST elevation and relative change of median power for total and each EEG frequency band (delta, theta, alpha and beta) in time windows H1, D1 and D2 in relation to B and scatterplots of statistically significant outcomes were depicted in Fig 6. There was a strong positive correlation between the ST elevation amplitude and relative change in alpha power in D1 (r = 0.85, p<0.05, Fig 6A). Also, relative change in beta power in H1 (r = 0.86, p<0.05, Fig 6B) and D1 (r = 0.90, p<0.05, Fig 6C) strongly correlated with ST elevation. Relative change in power of other frequency bands and total power in relation to power in B were not significantly correlated with ST elevation in any time window (r in range from 0.31 to 0.62, p>0.05).

## Discussion

### Main findings

In our current study, we used a model of AMI induced by intraperitoneal injection of isoprenaline (150 mg/kg). Onset of ischemia and necrosis was detected by ST segment elevation and infarction was confirmed by histologic examination of the myocardium for each animal in the study. This model of AMI was previously extensively validated for its construct validity in both functional and morphological aspects [22].

Our results have shown that experimentally-induced AMI affects cortical EEG recordings over the medial PFC as follows: 1) Beta band power was significantly increased both in the first hour (H1) as well as in the second to fourth hours (D1); 2) Theta band power was significantly increased in the first hour (H1); 3) Alpha band power was significantly increased between the second and fourth hours of recording; 4) All changes in band spectral power reversed to baseline levels in the recording made after 24hours; 5) Alpha and Beta band power changes were in significant correlation to the magnitude of ST elevation, which was used as a marker of infarct size, while theta band changes did not show statistically significant correlation.

This is, to the best of our knowledge, the first study to show the effect of isoprenaline-induced AMI on cortical EEG recordings and its dynamics.

### Medial PFC

It has been known for a long time that the medial PFC plays a major role in regulation of emotion, cognition, and executive functioning [27–30]. Studies in rodents have established medial PFC as one of key regions for regulation of the stress response and development of anxiety states [31].

The medial PFC also lies at the crux of cortical regulation of visceral functions and was even called the "visceral motor cortex" [32]. Functional studies involving stimulation/lessoning of medial PFC sub regions have worked out in detail the autonomic effects evoked from this region: respiratory frequency (phrenic nerve discharge frequency), cardiovascular (heart rate and arterial pressure) and metabolic rate are all significantly affected, showing the intimate connection of affective and emotional states with autonomic control provided by the medial PFC as an integrative “command” center connecting both autonomic and executive functions [33–35].

Also, epidemiological studies have shown that atherosclerosis, atrial fibrillation and heart failure lead to increased risk for dementia and that even subclinical decrease of cardiac output can lead to issues with cognitive function [36,37].

The results of previous studies could be ascribed to disturbances in medial PFC function and changes in EEG spectral power shown in our study make a step forward in connecting heart damage and medial PFC bioelectrical activity.

### What changes in EEG beta band power could tell us?

Higher frequencies of the EEG spectrum, such as beta frequency, increase in power during states of arousal [38, 39]. On the other hand, increases in beta power could also be induced by impaired emotional control and anxiety states [40, 41]. On a structural level, it was shown there is remodeling of neuronal networks in the medial PFC under the influence of stress [42]. Furthermore, according to findings of Banozic et al., anxiety like behavior can be demonstrated in a model of AMI [11]. In our current study the increase in beta band power was already significant during the first hour (H1) and remained increased during the whole four hours (D1) after isoprenaline administration. In light of this, the increase in beta frequency power observed in our study, could be related to the anxiety and arousal caused by necrosis/pain that occurs during AMI.

### What changes in EEG theta band power could tell us?

In light of related literature, the increase of theta band power recorded during our experiment could be linked to pain perception as well as anxiety which are both known to occur during AMI. There have been several studies in rodents focusing on the signature of pain in EEG recordings. A study by Le Blanc et al (2016) [43], focused on acute, inflammatory and neuropathic forms of pain and their effects on the PFC EEG signal. It was shown there is an increase in EEG spectral power over the PFC in all forms of pain studied [44]. Moreover, acute somatosensory pain showed an increase in theta band EEG power over S1. Increased PFC theta band power was also detected when rats with induced chronic neuropathic pain were faced with anxiogenic stimuli [45]. It is of note that cardiac pain occurring during AMI is a form of acute visceral pain known to cause significant anxiety. Indeed, prior research has implicated visceral pain as a cause of anxiety states [46]. On the other hand, there have been no prior reports regarding the EEG signature of visceral pain. Knowing that heart afferents connect to the PFC we may hypothesize that increase in theta power observed relates to the perception of visceral pain, and together with increased beta power, increased anxiety occurring during isoprenaline induced AMI.

### What changes in EEG alpha band power could tell us?

Previous studies correlated the increase in cortical alpha power with mental states of focused internal attention and cognitive effort [47]. Heightened alpha activity was also implicated in basic (memory, attention) as well as advanced cognitive functions (convergent and divergent thinking) [48–50]. Increase of alpha band power during D1 period found in our study may denote increased attention and cognitive vigilance. Thus, in light of these findings revealed by others neural signaling from intrinsic heart afferents stimulated by necrosis during isoprenaline-induced AMI could be manifested as increased focused attention- a favorable state for reaction of organism to imminent danger.

### Correlation of EEG changes with ST elevation and potential explanations

We correlated peak ST- segment elevation with the changes in the power spectrum. ST segment elevation represents the potential difference between ischemic and non-ischemic zones of myocardium caused by loss of cell membrane function in ischemic areas [26]. The magnitude of ST elevation is used as a marker for the size of myocardial infarction [25]. Potential mechanisms explaining the observed changes in EEG power of canonical band and the correlation with the magnitude of ST elevation in our current study are multiple but can be separated into two main segments: neural mechanisms and humoral mechanisms. The speed by which the change in EEG power follows onset of ischemia strongly implies a neural mechanism although humoral factors cannot be excluded. Namely, it is known that afferent inputs from the heart neural circuits are implicated in modulation of the CNS functions even under physiologic conditions [51]. Mechanical and hormonal information is transduced into nerve impulses by sensory neurons in the heart before being processed [52] and then sent to the CNS via the vagous nerve and dorsal columns spanning all the way to the medial PFC [53]. Also, the size of the ischemic area, as represented by magnitude of ST elevation influences the number of nervous fibers involved in signaling possibly explaining the correlation we observed. These anatomical connections represent a direct heart-brain axis neural link potentially explaining the EEG changes observed in our study.

### Limitations

We used the experimental model of isoprenaline induced AMI which is verified as a robust model widely used in different preclinical animal research and cited in other publications in this and other journals [22, 23, 54, 55]. We confirmed the occurrence of AMI by functional tests (ST segment analysis) and histological verification of myocardial necrosis. There is, however, a concern that isoprenaline itself could affect cortical EEG activity. This probability is lessened by highly hydrophilic structure of isoprenaline making it difficult to cross the blood brain barrier (BBB) [56]. Indeed, previous research of isoprenaline pharmacokinetics revealed a very low extraction in brain tissue, that can be attributed only to areas of brain devoid of the BBB [56]. The mPFC is a structure with an intact BBB making it probably very difficult for isoprenaline to have a significant direct effect. Moreover, isoprenaline has a very short plasma half-life of 2–4 minutes due to fast degradation by monoamine oxidase (MAO) and catechol-O-methyltransferase (COMT) enzymes [57] Additionally it does not bind to tissues and most of its presence is gone after 10 minutes [58]. Therefore, the early effect observed (1 hour) after the injection is also unlikely to be solely the effect of isoprenaline. In addition, some papers [59,60] indicate that isoprenaline has modulatory effects on the BBB. These effects of isoprenaline on the BBB are mediated by adrenergic receptors that are also stimulated by endogenous substances (adrenaline, noradrenalin etc.) secreted during the natural course of AMI. On the other hand, the indirect effects of peripheral beta receptor activation by isoprenaline on cortical activity cannot be excluded. However, increased sympathetic activity and adrenaline release due to myocardial necrosis is a hallmark of the natural course of acute myocardial infarction. Endogenous secretion also effects peripheral beta receptors thus causing similar effects to isoprenaline bringing this model somewhat closer to the naturally occurring phenomenon [55].

In our current study, we focused on changes in the EEG perceived during the acute faze post isoprenaline- induced AMI. However, we cannot exclude changes that could be found using longer recording periods. The use of the coronary artery ligation model of AMI was not an option for examining imminent effects of AMI in freely moving rats. Namely, the coronary artery ligation model of AMI has its advantages, but an important limitation is related to the extensive surgery necessary for establishing AMI and the use of general anesthesia. Myocardial ischemia starts before the end of the operation and even more time is necessary for the anesthetic to be metabolized upon sternum closing. This creates important biases concerning qualitative changes in EEG activity during the acute faze that use of a sham operated control group cannot reliably exclude. Our results contribute to further characterization of isoprenaline- model of AMI in conscious free-moving rats. Additionally, further research is feasible in other experimental models and in chronic faze of AMI. Future studies may include coronary artery ligation model of AMI with control group including thoracotomy without coronary occlusion.

Finally, we observed the natural course of AMI, without any interventions aiming to modify it. In the era of primary PCI (percutaneous coronary intervention) the natural course of AMI has dramatically changed from sudden cardiac death in favor of developing chronic heart failure (CHF) after (often multiple) AMI. Thus, further research of the heart brain connection is necessary on different, chronic heart disease models that would give us more insight into human morbidity in the primary PCI era.

## Conclusion

Our results demonstrate significant increases in median band power of beta, theta and alpha bands during experimental isoprenaline—induced model of AMI. Our results imply an influence of isoprenaline induced AMI on medial PFC activity possibly creating a heightened state of interoception which interrelates sensory, emotional and even cognitive states. These are the first findings to connect heart damage during AMI to disturbances in cortical bioelectrical activity.
